# An Essay on the Mode by Which Constitutional Disease Is Produced from the Inoculation of Morbid Poisons

**Published:** 1817-10

**Authors:** 


*An Essay on the Mode by which Constitutional Disease is produced from the Inoculation of Morbid Poisons.* By CHARLES SALT, M. C. S. *Octavo, pp.* 88 *London,* 1817.

THIS little volume is characterized by modesty, good taste, and no despicable share of ingenuity. Without vouching for the infallibility of Mr. Salt's theoretical views, we conceive that the scope of his doctrines tends to a useful and practical point; which is more than can be said of first Essays in general. But we shall proceed to lay a full analysis of the work before our readers, leaving them to judge for themselves.

CHAP. I. Mr. Salt confines his remarks to the introduction of those morbid poisons through the *external* part of the body which come under the following definition, viz. some animal secretions produced under the influence of disease, and capable of propagating a specific *constitutional* disease by inoculation.

Mr. S. does not believe that morbid poison introduced from one animal into the absorbents of another, will *directly* produce constitutional effects ; for either it is innocent if absorbed in this state, or becomes so in its passage through the absorbents. He thinks, that those specific effects are produced by a fluid secreted by the arteries of the inoculated animal, very similar to the poison applied to them.

A *morbid* poison applied to an abraded surface has a specific power inherent in it, of exciting a new secretory action in the very contiguous arteries, by which a poisonous fluid is secreted, which, when absorbed from the part, affects *directly* by such absorption, and any other individual *indirectly* by a repetition of this acquired secretory process. This process is invariably slow, preceded and attended by local inflammation. The following are the principal reasons that have induced Mr. S. to offer these opinions. 1st. He is not aware of any reason why the deposited *morbid* poison should not be speedily absorbed, instead of remaining, as in some cases of syphilis, a whole month without any absorption taking place. 2d. It appears almost miraculous, that a poison can lie dormant for a very considerable time in an organised part; whereas, by an easy mode of explanation, it does not lie dormant, but speedily gives an impulse to the contiguous arteries. This impulse, it is true, does not produce immediate inflammation and secretion in these vessels; each poison also varies in the length of time required to produce this effect. But in the case of a mechanical injury, as a bruise for example, the part injured frequently remains several days without pain or inflammation; yet, some time after its apparent restoration, inflammation and a secretion of pus ensue. In incised wounds, actual secretion of pus is not produced in less than from thirty to sixty hours. 3. Out of four persons carefully inoculated from the same pustule, two may have local inflammation and secretion followed by the usual constitutional effects; but the others may have neither the one nor the other. The function of the absorbents cannot be thus inactive and uncertain; the difficulty vanishes if we admit that the absorption of the deposited poison is harmless. — Should this explanation be established by proof, we must infer that nothing is to be feared from the absorption of *morbid* poison, if the suppuratory process in the contiguous arteries of a part in which the poison has been inoculated is prevented. He supports his theory by the following arguments.

1st. " After inoculation, simple absorption of the deposited fluid is found incapable of producing constitutional disease, without the intervention of a local secretory process."

This argument requires to be strengthened by proof to the full as much as the theory it is meant to support. It is in fact, merely a repetition, in nearly similar words of the original hypothesis ; Vide page 2. " Briefly — it appears to me, &c."

2d. " Constitutional effects (as far as experience allows of a decision) may be always prevented by excision or destruction of the contiguous organized structure, antecedently to the local suppuratory process.

3d. " From general experience, where the primary suppuration or secretion goes through its regular process, the constitutional disease, among individuals capable of receiving the complaint, may be always expected to follow."

Chap. II. Mr. Salt proceeds to take a view of the respective functions of the arteries and absorbents, in reference to the uniformity and expedition with which they perform their office in health. He knows of no instance (supposing function is unimpaired) where fluid bodies remain any great length of time intimately applied to the absorbents without being absorbed, except it be so in the case of morbid poisons; even these he considers no exceptions to the general rule. He supports this opinion by the consideration that these vessels have not a power of selecting the substances they absorb; for we find that bile is frequently absorbed and carried into the mass of blood, where it produces that yellow colour observable upon the eyes and skin of persons suffering under jaundice. Mr. Salt therefore concludes, that the absorbents are regulated in their action by one general law; viz.

"That of absorbing all fluid bodies intimately applied to them in a moderate time, without exception; and that the only difference of function in various parts arises from their possessing a greater or less proportion of these vessels."

*Arterial suppuratory Secretion.* Mr. Salt has shown that the function of the absorbents is a *healthy* function, whereby the body is assisted in its growth, and divested of superfluous and extraneous matter. He now considers a *diseased* function of a very different kind; a function not in existence in a healthy body, and whose very presence constitutes disease; namely, the process of *arterial secretion,* which is provided by Nature, either for the restoration of injured parts, or under the immediate influence of constitutional disease. The healthy secretions are performed by certain arteries, endowed with a secreting power in the original formation of the body; but the arteries of every portion of the system seem capable of assuming the function of secretion under the influence of disease. "Nature," says Mr. S. "always reluctant to admit disease, makes, when it is practicable, an effort to avoid the process of suppuration in the first instance after the infliction of injuries." Suppuration must, however, occasionally ensue; but this process is admitted with reluctance, and is performed with a degree of tardiness. A very long period is sometimes required to produce suppuration after the insertion of extraneous bodies; hence, it is not extraordinary, that morbid poisons, each according to its specific nature, should require a certain time to produce a local suppuration or secretion in the part to which they are applied. Mr. S. considers this as by no means improbable: it appears to him to be a fact in perfect accordance with the other phænomena observable in the process of suppuration. But he thinks it a monstrous stretch of credulity to believe, that after the insertion of morbid poisons, the healthy function of the absorbents of the part should be suspended, a week or two, and then acquire vast energy and activity. He thinks that the first accords with the common laws of morbid secretion: but the latter is of a complexion wholly at variance with all the known laws of function.

CHAP. III. We find, then, that the arteries, having received their specific impulse by the inoculation of *morbid* poisons, after some time secrete a fluid *sui generis*, which is capable of producing the same disease, under certain circumstances, in others. There is another class of poisons produced by *healthy* secretion in certain animals, chiefly for the purpose of self-defence; such are the poisons of the various kinds of serpents, &c. When these poisons are introduced into the human body by inoculation, the absorbents, according to their common law, at once begin to convey them to the mass of blood; and unless the progress of the poison be interrupted by ligature, or excision of the part, the patient soon dies. This fact seems to afford a strong reason for supposing that *morbid* poisons, in common with others, cannot remain long exposed to the action of the absorbents without absorption ensuing. The morbid poisons *appear*, however, to deviate from the general law, and a certain time is required to elapse before their effects are apparent: and farther, there must be a local secretion of a fluid in the part to which they were applied, *antecedently to the production of the constitutional effects*. In the majority of cases of hydrophobia, indeed, there has been hitherto no absolute demonstration of the local secretion of such a fluid. But Mr. Salt thinks, such a local secreting process does always ensue in this disease, although it has not generally been detected. Dr. Hamilton, in his work on Hydrophobia, says,

"It is rare if the cicatrix fails to reinflame at a certain time after incarnation, exhibiting evident marks of absorbed infection."

A volume might be filled by quotations of cases, in proof of local pain ensuing at the time of the commencement of constitutional disease; Mr. Salt has given several extracts on the subject of Hydrophobia, from the Medical Commentaries, and from Dr. Hamilton's remarks, that serve to shew how generally reinflammation occurs in the part originally bitten, attended by pain in the cicatrix, immediately before the symptoms of the disease appear. It should seem from these cases, that excision of the wounded part may be, and frequently is, attended with complete success, if it be performed *before* constitutional disease commences, though at any period after the part has been bitten. Dr. Darwin thought it probable, that even after the appearance of hydrophobia it might be cured, if the patient had been bitten in a part which could be totally cut away, as a finger. It is certainly an object of the highest importance to investigate every circumstance connected with this disease; for, when it is acknowledged that the disease is *incurable*, surely it becomes the duty of every man to push the means of prevention to the utmost extent.

In the small-pox, in common with other morbid poisons, there is this difference between the matter reproduced by local secretion, before its removal from the body of the patient, and subsequent to such removal: in the former state, it infects, by direct absorption, the person in whom it is secreted; and in the latter, it possesses no such property of infecting that, or any other individual, by direct absorption, as its power of that kind ceases on its removal from the body in which it was generated. Mr. Salt is aware that an objection may be made to the supposition, that "a secretion, while enclosed in the part in which it was secreted, may possess a property which it is unable to retain after its removal," on the ground of absurdity. He, however, observes, that many healthy secretions undergo precisely similar changes from exposure to air, and removal from the body.

"The blood, for example, while circulating in the living animal, possesses certain known properties essential to the continuance of life: remove it from the body, and in a very short time (perhaps a few seconds) it loses the peculiar powers and properties it possessed when in a state of circulation. If such blood be returned into the veins of an animal, it becomes, from the change it has undergone, a mere extraneous matter, no longer capable of the performance of its usual functions."

Mr. Salt gives a very ingenious illustration of the mild variety of the small-pox from inoculation, by comparing it to a variety of a flower, only to be obtained by a peculiar mode of propagation. The flower, propagated by a slip, has the *essential* qualities of another produced from seed, and all the perfect character of its species; the latter has the same; but the individual beauty of a particular flower is not to be expected from such plants as are subsequently produced by its seed. To preserve the variety, they must all be obtained by the peculiar mode of propagation by slips. That the mild variety in the disease of small-pox from inoculation, compared to the original disease, is, like the flower, a result only to be obtained by a peculiar mode of propagation, appears undeniable; we still, however, find the important specific qualities in each poison secreted in the respective diseases perfect and unimpaired. In maintaining then the character of its species under *all* modes of propagation, and being distinguishable in some as a variety only, the disease produced by the poison is very analogous to what has been instanced in the flower.

Part II.—Chap. I. It is essentially characteristic of the diseases produced by inoculation of variolous or vaccine matter, by either applying it after the cuticle has been abraded, or depositing it in the interior of a very small incised wound, that a local secretion, in the part where the poison was inserted, should precede the constitutional effects. No immediate effects follow the application of the poison; the small wound in many instances appears quite restored by the adhesive process; but every practitioner is aware, that unless a subsequent local inflammation ensues, he is not to expect the constitutional effects. The relative progress of the *pustule* in small-pox, occasioned locally by inoculation, and of those pustules produced subsequently upon the surface of the body, is curious, the latter never appearing until the former shall have been sufficiently matured to have had a secretion in its interior.

Among the many thousands of persons who have been inoculated for small-pox, and in whom no local symptoms took place, there must surely, Mr. Salt observes, have been many (considering the number and activity of the absorbents), in whom absorption of the matter occurred, in the state in which it was conveyed from the original person; yet no such constitutional effects ensued. He thinks it probable, that in such cases the matter was absorbed before it had imparted the impulse to the arteries of the part which gives them their secretory power. It is indeed difficult to believe that the absorbents are thus inactive and uncertain; but the difficulty vanishes, if we admit that the absorption of the deposited poison was in such cases harmless.

CHAP. II. *Syphilis.* There is a considerable interval between cause and effect in this disease, which renders its investigation easier, and the result more satisfactory, than is the case in the inferences drawn from the history of the inoculation of any other morbid poison. It sometimes happens that, after the deposition of the poison, no local effect is apparent until five or six weeks have elapsed. *After* the appearance of a chancre, but in no instance *previously*, the usual constitutional consequences arise, as ulcerated sore throat, nocturnal pains, &c. produced, as it would appear, by the newly secreted poison from the chancre being carried into the constitution by the absorbents.

"Why (asks Mr. Salt), in this case, if the poison in the state it was introduced, was alone sufficient to produce constitutional effects; why might it not have happened that those effects should have preceded, or been contemporary with, the appearance of the chancre? Yet no such circumstance ever occurred."

Mr. Salt gives several cases, where the application of caustics to chancres as fast as they appeared, has, by destroying them, and consequently preventing suppuration, totally cured syphilis without any constitutional effects following in any instance. Hunter, in his work on Syphilis, says, that he has dissected out chancres; and states, from experience, that their destruction will prevent constitutional disease. It would not, however, be safe to put implicit confidence in this method of cure, as we cannot ascertain, at least in the present state of our knowledge, the precise point of time at which the absorption of the secondary secretion shall have ensued.

We have thus given a full analysis of Mr. Salt's little work, which is creditable to his talents, to his taste for composition, and to his modesty and good sense as an Author.

PART

